# 538. Human neutrophil gelatinase-associated protein (N-gal) and proinflammatory cytokines as biomarkers of disease severity in children with Hemolyitic Uremic Syndrom (HUS) and Shigatoxin-producing E.coli (STEC) infection.

**DOI:** 10.1093/ofid/ofac492.591

**Published:** 2022-12-15

**Authors:** Analía Toledano, Ana Caratozzolo, Romina Lanfranchi, Mayra Martinez, Marian Chacoff, Laura Talarico, María M Contrini, Eduardo Luis Lopez

**Affiliations:** Pediatric Infectious Diseases Program, Hospital de Niños "Dr. Ricardo Gutiérrez", Universidad de Buenos Aires, Buenos Aires, Ciudad Autonoma de Buenos Aires, Argentina; Pediatric Infectious Disease Program, Hospital de Niños Ricardo Gutiérrez, Universidad de Buenos Aires, Buenos Aires, Ciudad Autonoma de Buenos Aires, Argentina; Hospital de Niños Ricardo Gutierrez, CABA, Ciudad Autonoma de Buenos Aires, Argentina; Hospital de Niños Ricardo Gutierrez, CABA, Ciudad Autonoma de Buenos Aires, Argentina; Hospital de Niños Ricardo Gutierrez, CABA, Ciudad Autonoma de Buenos Aires, Argentina; Hospital de Niños Ricardo Gutierrez, CABA, Ciudad Autonoma de Buenos Aires, Argentina; Pediatric Infectious Disease Program, Hospital de Niños Ricardo Gutiérrez, Universidad de Buenos Aires, Buenos Aires, Ciudad Autonoma de Buenos Aires, Argentina; Hospital de Niños Ricardo Gutierrez, CABA, Ciudad Autonoma de Buenos Aires, Argentina

## Abstract

**Background:**

Hemolytic Uremic Syndrome (HUS) is a complication of Shigatoxin-producing *E.coli* (STEC) infection which presents a characteristic triad of microangiopathic hemolytic anemia, thrombocytopenia, and acute renal failure. Preliminary *in vitro* and experimental animal studies demonstrated that Shigatoxins (STXs) induce the secretion of proinflammatory cytokines. Human neutrophil gelatinase-associated protein (N-gal) has been reported as a marker of acute kidney injury. Dosing serum levels of IL-8, TNF-α, IL-6, IL-1β and N-gal in children with STEC-associated infection and HUS would allow to establish the role of these cytokines as biomarkers of renal injury and severity

**Methods:**

Prospective study during 2017 – 2020 was performed; three groups of patients < 18 years were included: bloody diarrhea (BD), HUS requiring dialysis (HUSD) and HUS and no dialysis requirement (HUSND), all of them with presence of STX in stool. Blood samples were collected at diagnosis (t1) and at 7 days (t2). An immunoassay was used for detection of TNF-α, IL-1β, IL-6, and IL-8 (Bio-Plex Pro Human®, BioRad). An immunoassay (Anti-Lipocalin-2) was used for N-gal detection

**Results:**

Forty-nine children were admitted; 22 (49%) were male. Median age was 24 (IQR 13-36) months. Fourteen patients with BD, 24 patients with HUS: 12 HUSD and 12 HUSND. Eleven healthy children (HC) were included. At diagnosis, higher IL-8 values were found in HUSD vs. BD (p=0.0004), HUSND (0.003) and HC (p= 0.0043). Higher TNF-α values were detected in HUSD vs. BD (P= 0.0001), HUSND (p= 0.0011) and HC (p= 0.0002). Higher TNF-α values were found in patients with BD vs HC (p= 0.0055). HUSD exhibited higher IL-6 values as compared with BD (p= 0.0145) and HC (0.0106). N-gal concentrations were higher in HUSD vs. BD (P= 0.0120), HUSND (P= 0.0166) and HC (p= 0.0049). A decrease in IL-8 was observed in HUSD between t1 and t2 (p=0.0357). (Figure 1)

**Figure:**
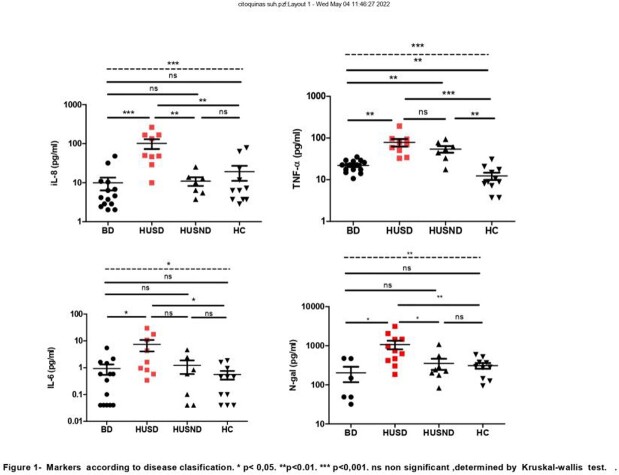

**Conclusion:**

Interleukin 8, IL-6, and N-gal may be considered as potential serological markers of renal damage and dialysis requirement. TNF-α concentrations are already increased in BD without HUS complications

**Disclosures:**

**All Authors**: No reported disclosures.

